# Acceptance and experience of digital dental technology, burnout, job satisfaction, and turnover intention for Taiwanese dental technicians

**DOI:** 10.1186/s12903-022-02359-z

**Published:** 2022-08-11

**Authors:** Tang-Yun Teng, Ju-Hui Wu, Chen-Yi Lee

**Affiliations:** 1 Department of Oral Hygiene, College of Dental Medicine, Kaohsiung Medical University, Kaohsiung, Taiwan; 2 Division of Family Dentistry, Department of Dentistry, Kaohsiung Medical University Hospital, Kaohsiung, Taiwan; 3 Department of Medical Research, Kaohsiung Medical University Hospital, Kaohsiung, Taiwan

**Keywords:** Burnout, Digital dental technology, Dental technicians, Job satisfaction, Turnover intention

## Abstract

**Background:**

Digital dental technology (DDT) has progressed and been introduced to Taiwan in the recent years, gradually changing the industry ecology. Many studies have demonstrated that DDT is more accurate and faster than conventional dental technology. However, there is a paucity of research exploring dental technicians’ perspectives on digital dental techniques, and their burnout, job satisfaction, and turnover intention.

**Methods:**

This cross-sectional survey with convenience sampling was conducted at the conference venue of the Taiwan Association of Dental Technology to investigate the perspectives of dental technicians. We used the snowballing method in this study; two sampling methods were adopted, a convenience sampling of dental technicians to complete a survey, followed by asking the survey participants of the convenience sample to invite their colleagues to participate in the online survey. The survey questionnaire included questions on demographics, work-related information, acceptance and experiences of dental technicians toward DDT, occupational burnout, job satisfaction, and turnover intention. Regression models were used to determine the predictors of job satisfaction and determinants of turnover intention.

**Results:**

In total, 341 valid questionnaires were obtained. Overall, the participants reported long working hours (95.5%), positive score on the DDT acceptance scale, moderate job satisfaction, higher personal burnout, and work burnout, along with lower over-commitment. Among them, 32.9% and 28.2% reported the intention to leave their organization and profession, respectively. The stepwise multiple regression model revealed that higher work burnout decreased job satisfaction, while higher DDT acceptance and position as employer increased job satisfaction. The binary logistic regression models revealed that geographical area of workplace, work burnout, and job satisfaction were significant predictors of turnover intentions.

**Conclusions:**

Many Taiwanese dental technicians reported turnover intentions and higher burnout. With the trend of digitalization in the dental industry, even though most dental technicians had a positive outlook toward DDT, its influence on job satisfaction appears limited. Retaining good and professional talents required of a dental technician is crucial, especially as Taiwan’s dental care becomes increasingly specialized. Strategies for improving the work environment and occupational health of dental technicians should thus be the focus of future studies.

## Background

Over the past few years, digital technologies have significantly contributed to clinical procedures and laboratory methodologies in dentistry. The introduction of intraoral scanners and advanced fabrication processes such as computer-aided design and manufacturing (CAD/CAM) technologies and 3D printing has enabled the substitution of conventional metal frameworks [1]. Compared to conventional dental technology (CDT), digital dental technology (DDT) has increased the time efficiency of laboratory fabrication [2, 3]. The digital workflow has demonstrated better clinical efficiency when considering impression time, patient preference, and time efficiency [3]. Most British and Irish dental technicians reported using some form of CAD/CAM technology in their workflow [4]. Global digitalization has changed the dental industry and the working content of dental technicians in Taiwan. A previous study investigating the acceptance of CAD/CAM among Taiwanese dental technicians reclaimed that CAD/CAM was the future of the dental industry and would likely replace most of the manufacturing processes currently undertaken by dental technicians. [5]

Few studies have focused on occupational diseases or injuries of dental technicians in dental laboratories. Previous studies reported that dental technicians exposed to sandblasting have a higher risk of pneumoconiosis [6] and also have an increased risk of developing hand eczema owing to frequent exposure to uncured (meth)acrylates and handwashing [7]. A high prevalence of work-related musculoskeletal disorders [8] and the infection risk of dental impressions [9] have also been reported.

Dental technicians are an important part of dental teams; they are licensed dental auxiliaries in many countries, including Taiwan [10]. However, compared to dentists or dental hygienists/therapists, few studies have investigated the occupational health issues of dental technicians [11]. In the UK, a study revealed that even though dental technicians showed a high level of job satisfaction, < 50% of them felt adequately valued as individuals in the dental team and as a professional group [12]. Another common issue of occupational health is burnout, which is a prolonged response to chronic emotional and interpersonal stressors in a job [13]. It is common among individuals with people-centered occupations [14]. A previous study in Taiwan showed a moderate burnout level among dentists and a high burnout level among dental assistants in the context of national health insurance. The most stressful event for dental staff was identified to be “managing medical disputes or lawsuits” [15]. Likewise, some studies have revealed that managing anxious or difficult patients is associated with burnout in dental staff [16, 17]. Moreover, previous studies in medical settings have shown that burnout and job satisfaction are both associated with turnover intention [18–22].

In Taiwan, dental technicians predominantly work in laboratories, and their burnout levels may differ from those of other dental team members. Furthermore, the industry’s trend toward digitalization may impact their working contents and occupational health; however, to our knowledge, no study has explored these issues. The purpose of this study was twofold: (1) to explore the working characteristics and DDT experiences of dental technicians, and their association with job satisfaction, and (2) explore the factors associated with turnover intention among Taiwanese dental technicians.

## Methods

### Study design and participants

The Dental Technicians Association was not able to provide the member list due to the Personal Data Protection Act. A randomized sampling approach was impossible; therefore, two nonrandomized sampling approaches were adopted in this study. This cross-sectional study was first conducted on May 25, 2019, at the conference venue of the Taiwan Association of Dental Technology. When the attendees who visited the stall were confirmed by the researchers as licensed and practicing dental technicians, the researcher informed them of the study's purpose and the dental technicians who agreed to participate chose to complete the questionnaire on paper or online. This study did not provide rewards for participation; for improving their willingness to complete the questionnaire, we allowed the participants the choice of anonymity. A convenience sample was collected at the conference venue, and the link to the online survey was provided to the participants to invite individuals unable to attend the conference by the snowballing method. After the conference, there were further inquiries from potential volunteers about participation in the online survey; therefore, the online data was collected till September 21, 2019.

The online survey was set up in a way that the participants were required to type their email addresses, each email could only complete the questionnaire once to avoid duplicate responses, and any other possible duplicates were contacted via email. The inclusion criteria were dental technicians who had passed the national examination, with their current job in a dental laboratory, and were responsible for dental technology for at least one year. A total of 221 completed questionnaires were received on paper, and the remaining 127 were submitted online. After excluding the duplicates and respondents with seniority of < 1 year, 341 valid questionnaires were included in this study (217 papers and 124 online).

### Ethical approval and consent to participate

The research protocol was approved by the Institutional Review Board of Kaohsiung Medical University Hospital (KMUIRB) (KMUHIRB-E[I]-20190094). All methods were carried out in accordance with relevant guidelines and regulations. The use of altered informed consent was approved by the ethics review board due to the anonymous and online nature of the study design.

### Instruments

The structured questionnaire included demographic characteristics (such as sex, age, and education), working characteristics (such as geographical area of workplace, job position, working hours, salary, and seniority), experiences of DDT use (such as work content, job duties, types of DDT used, and number of DDTs learned), DDT acceptance, occupational burnout, job satisfaction, and turnover intention.

#### DDT acceptance

**Fig. 1:**
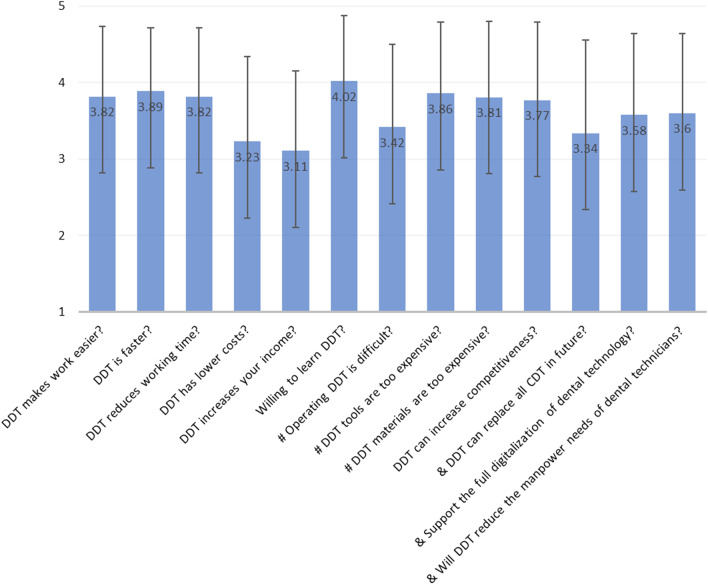
Attitudes of the participants toward DDT. ^#^items regarding disadvantages, reverse scored when a sum is calculated. ^&^items regarding future trends. The items without any symbol refer to advantages

The DDT acceptance scale consisted of 13 items that were developed by the authors, based on literature review, and a few in-service dental technicians (Fig. 1). The scale included seven items related to the advantages of using DDT, three related to disadvantages, and three regarding future trends. The items were answered using a 5-point Likert scale ranging from 1 (strongly disagree) to 5 (strongly agree), and a sum score range of 13–65 indicated that DDT acceptance was obtained. Internal consistency for the scale in this sample was acceptable (Cronbach’s α = 0.77).

#### Occupational burnout

Occupational burnout was measured using the Occupational Burnout Inventory (OBI) developed by Yeh et al. [23]. The present study adopted the subscales related to personal burnout, work burnout, and over-commitment, each containing five items. The subscales of personal and work burnout were modified by Yeh et al. [23] from the Chinese version of the Copenhagen Burnout Inventory [24]. The over-commitment subscale was modified by Yeh et al. [23] from the Chinese version of the Effort-Reward Imbalance Model Questionnaire. The response scale ranged from 0 (never) to 4 (always), and the sum scores of each subscale were multiplied by 5; therefore, a full score ranging from 0 to 100 was obtained. The Cronbach’s α of the subscales ranged from 0.84 to 0.90.

#### Job satisfaction

Job satisfaction was measured using five items adopted in a study by Judge et al. [26]; those items were taken from the Brayfield-Rothe [25] measure of job satisfaction [25, 26]. The five items taken by Judge et al. [26] were as follows: “I feel fairly well satisfied with my present job”; “Most days I am enthusiastic about my work”; “Each day of work seems like it will never end” (reverse scored); “I find real enjoyment in my work”; and “I consider my job rather unpleasant” (reverse scored). The response scale ranged from 0 (strongly disagree) to 10 (strongly agree), and the sum score ranged from 0 to 50 [26]. In this study, the items were translated into Chinese by a bilingual translator, and the Cronbach’s α was 0.88*.*

#### Turnover intentions

Turnover intentions were measured as described by Blau and Lunz [27], and participants were asked to indicate their agreement with the following items: “I intend to leave the organization” and “I think about leaving the dental technician profession.” The items were rated on a four-point scale (1 = strongly disagree, 4 = strongly agree) [27].

### Data analysis

All data were analyzed using the IBM Statistical Package for Social Sciences for Windows version 20.0 (SPSS Inc., Armonk, New York, USA). A significance level of 0.05 was used for all statistical analyses. Differences among sociodemographic characteristics, workplace characteristics, and DDT experiences in job satisfaction were assessed using an independent sample t-test or one-way analysis of variance (ANOVA) when the scores were normally distributed; similar comparisons in terms of turnover intentions were assessed using Chi-square test. The correlations between the DDT acceptance, burnout, job satisfaction, and turnover intentions were assessed using Pearson’s correlation coefficients. Stepwise multiple linear regression analysis was conducted to determine the predictors of job satisfaction among dental technicians. Regarding determinants of turnover intentions, the two items were divided into binary variables (agree or disagree) for further analysis using logistic regression models.

## Results

### Sample characteristics, working characteristics, and turnover intentions

In this study, most participants were male (n = 213, 63.6%) and most were aged between 20 and 29 years (n = 233, 68.5%); the mean age was 29.11 years (SD = 6.76). Most of the participants reported that their highest educational level was junior college (n = 201, 59.3%), followed by college or higher level (n = 117, 34.5%); most reported the geographical area of workplace to be Taichung (n = 97, 28.6%), followed by Taipei metro area (n = 93, 27.4%) and Kaohsiung (n = 86, 25.4%). Regarding work characteristics, most participants were employees (n = 315, 94.3%) who worked 40–59 h per week (n = 280, 83.6%). Most participants had work experience of < 10 years (n = 297, 87.1%), with a mean of 6.38 years (SD = 6.22), and an income of < 40,000 NT dollars per month (n = 275, 81.7%). Regarding turnover intentions, 71.7% (n = 241) of participants reported that they did not have the intention to leave the profession, and 67.1% (n = 226) reported that they disagreed with the intention to leave the organization (Table 1).

**Table 1:** Sample characteristics, work characteristics, and turnover intentions of the participants (n = 341)

Variables	Valid responses (n)	%
*Reply means*		
Paper	217	63.6
Online	124	36.4
*Sex*		
Male	213	62.8
Female	126	37.2
*Age group (years)*		
20–29	233	68.5
30–39	79	23.2
≥ 40	28	8.2
*Highest education level*		
Senior/vocational high school or lower	21	6.2
Junior college	201	59.3
College or higher	117	34.5
*Geographical area of workplace*		
Taipei metro area	93	27.4
Taichung city	97	28.6
Kaohsiung city	86	25.4
Taoyuan city	35	10.3
Other cities	28	8.3
*Job position*		
Employer	19	5.7
Employee	315	94.3
*Working hours (per week)*		
< 40	15	4.5
40–49	164	49.0
50–59	116	34.6
≥ 60	40	11.9
*Salary (per month, NT dollars)*		
≤ 23,000 ^#^	15	4.5
23,001 – 30,000	101	30.0
30,001 – 40,000	159	47.2
40,001 – 50,000	33	9.8
≥ 50,001	19	8.6
*Work experience (years)*		
<10	297	87.1
≥ 10	44	12.9
*Intention to leave profession*		
Strongly disagree	59	17.6
Disagree	182	54.2
Agree	73	21.7
Strongly agree	22	6.5
*Intention to leave the organization*		
Strongly disagree	68	20.2
Disagree	158	46.9
Agree	83	24.6
Strongly agree	28	8.3

^#^: 23,100 NT was the minimum wage of 2019 as stipulated by the Taiwan government

### Work contents and DDT-related experiences

Regarding work contents, fixed partial dentures were the most frequent (61.3%), followed by removable dentures (32.6%), and digital fixed partial dentures (27.0%). Most participants reported that their job duties were CDT (70.4%), while others were responsible for DDT or both. CAD/CAM was the most popular DDT (62.8%), and only 85 participants (24.9%) reported that they had no experience in using any type of DDT (Table 2).

**Table 2:** Work contents and DDT-related experiences

Variables	Valid responses (n)	%
*Work contents (multiple choices)*		
Fixed partial dentures	209	61.3
Removable dentures	111	32.6
Orthodontic appliances	39	11.4
Digital fixed partial dentures	92	27.0
Digital removable dentures	16	4.7
Digital orthodontic appliances	20	5.9
Others	8	2.3
*Job duties*		
CDTs only	240	70.4
DDTs only	49	14.4
Both	52	15.2
*Types of DDT used (multiple choices)*		
CAD/CAM	214	62.8
Optical intra-oral scanner	102	29.9
3D printing (resin)	95	27.9
3D printing (wax type)	57	16.7
Others	1	0.3
*Number of DDTs learned*		
0	85	24.9
1	127	37.2
2	70	20.5
3	35	10.3
4	24	7.0

### DDT acceptance, burnout, job satisfaction, and turnover intentions

Figure 1 shows the participant's responses to DDT acceptance. Overall, the participants were willing to learn DDT, had positive attitudes toward DDT efficiency, and negative attitudes toward cost and price. The average sum score of the DDT acceptance scale was 43.10 (SD = 5.958), ranging from 25 to 57. The average job satisfaction was 26.09 (SD = 7.617), ranging from 0 to 47. The average scores of personal burnout, work burnout, and over-commitment were 51.13 (SD = 21.628), 46.56 (SD = 21.550), and 39.41 (SD = 19.473), respectively, with scores ranging from 0 to 100.

The Pearson correlation matrix showed that DDT acceptance (r = 0.198, *p* < 0.001), personal burnout (r = − 0.595, *p* < 0.001), and work-related burnout (r = − 0.638, *p* < 0.001) were significantly correlated with job satisfaction (Table 6). Moreover, DDT acceptance (r = − 0.185, *p* = 0.001), personal burnout (r = 0.495, *p* < 0.001), work-related burnout (r = 0.531, *p* < 0.001), and job satisfaction (r = − 0.623, *p* < 0.001) were significantly correlated with the intention to leave the organization. Furthermore, DDT acceptance (r = − 0.226, *p* < 0.001), personal burnout (r = 0.390, *p* < 0.001), work-related burnout (r = 0.431, p < 0.001), over-commitment (r = 0.178, *p* = 0.001), and job satisfaction (r = − 0.468, *p* < 0.001) were significantly correlated with the intention to leave the profession.

### Predictors of job satisfaction

An independent sample t-test revealed that sex (t = − 0.125, *p* = 0.901) and seniority (t = − 0.852, *p* = 0.395) had no significant effect on job satisfaction; however, employers showed a significantly higher job satisfaction than employees (t = 3.342, *p* = 0.001). One-way ANOVA showed that age group (F_2, 334_ = 0.049, *p* = 0.953), highest educational level (F_2, 333_ = 0.678, *p* = 0.508), income (F_4, 329_ = 0.982, *p* = 0.418), geographical area of workplace (F_4, 331_ = 1.064, *p* = 0.374), and job duties (F_2, 335_ = 1.678, *p* = 0.188) had no significant effect on job satisfaction; however, dental technicians with fewer working hours per week had higher job satisfaction (F_3, 328_ = 4.576, *p* = 0.004) (Table 7).

Significant univariate variables were further analyzed using stepwise multiple regression, which showed no collinearity among the variables (VIF = 3.867, CI = 18.889). The model revealed that higher work burnout decreased job satisfaction (*β* = − 0.650, p < 0.001), while higher DDT acceptance (*β* = 0.174, *p* < 0.001) and position as an employer (*β* = 0.147, *p* < 0.001) increased job satisfaction (Table 3).

**Table 3:** Predictors of job satisfaction among dental technicians

Variables	B	S.E	*β*	t	*p*-value
Intercept	26.163	2.389		10.950	< 0.001
Work burnout	− 0.219	0.014	− 0.650	− 15.974	< 0.001
DDT acceptance	0.220	0.051	0.174	4.279	< 0.001
Employer	4.957	1.376	0.147	3.603	< 0.001

Adjusted R^2^ = 0.496

Adjusted for working hours (per week), number of DDTs used, and personal burnout

### Determinants of turnover intentions

With regard to the intent to leave the organization, the chi-square test revealed that only geographical area (χ^2^ = 10.358, *p* = 0.035) showed a significant difference; the other variables were non-significant (Table 8). Regarding the intention to leave the dental technician profession, highest educational level (χ^2^ = 9.334, *p* = 0.009) and geographical area of workplace (χ^2^ = 18.431, *p* = 0.001) showed significant differences, and the other variables were non-significant (Table 9).

Significant univariate variables were further analyzed to explore predictors of turnover intention based on the results of the binary logistic regression models (Tables 4 and 5). The results showed that geographical area of workplace had a variable effect on both types of turnover intention. Moreover, individuals with higher job satisfaction were negatively related to turnover intentions, whereas those who had higher work burnout were positively related to turnover intentions. Furthermore, participants with educational level of junior college showed lower intention to leave the profession (Table 5).

**Table 4:** Determinants of turnover intention among dental technicians—Intention to leave organization

Variables	Intention to leave organization		
	aOR	95% CI	*p*-value
Geographical area			
Taipei metro area (ref.)	1		
Taichung city	0.904	0.393–2.079	0.812
Kaohsiung city	1.507	0.649–3.499	0.340
Taoyuan city	6.290	2.118–18.685	0.001
Other cities	1.257	0.366–4.317	0.717
Work burnout	1.038	1.019–1.058	< 0.001
Job satisfaction	0.811	0.756–0.870	< 0.001
Pseudo R^2^	0.380		

Adjusted for education level, personal burnout, over-commitment, and DDT acceptance

**Table 5:** Determinants of turnover intention among dental technicians—Intention to leave profession

Variables	Intention to leave profession		
	aOR	95% CI	*p*-value
Geographical area			
Taipei metro area (ref.)	1		
Taichung city	0.334	0.147–0.758	0.009
Kaohsiung city	1.265	0.574–2.787	0.560
Taoyuan city	1.795	0.670–4.804	0.244
Other cities	0.998	0.342–2.912	0.996
Highest educational level			
Senior/vocational high school or lower (ref.)	1		
Junior college	0.301	0.095–0.953	0.041
College or higher	0.871	0.266–2.852	0.819
Work burnout	1.021	1.004–1.038	0.018
Job satisfaction	0.896	0.849–0.945	< 0.001
Pseudo R^2^	0.238		

Adjusted for personal burnout, over-commitment, and DDT acceptance

## Discussion

Taiwan's dental medical division system commenced later than other developed countries, and the process of division of labor is controversial. Traditionally, the training of dental technicians is a Master-Apprentice system. Since the implementation of the "Physicians Act" in 1975 (which included dentists in the standard), some dental mold technicians are still engaged in medical practices such as filling or treatment, which formed the "secret doctor" controversy. The first official dental technology educational institution was established in 1981. After being questioned and opposed by dental physician groups at that time, and under the long-term wrestling and running-in of various interest groups, dental technicians were finally positioned as "not allowed to engage in any intraoral care work;" the "Dental Technicians Act" was passed in 2009, which included the management of dental technicians with different training backgrounds. The special historical context has caused today's dental technicians to be a relatively closed-off and self-protected group of professionals that are not easily referred by patients or the general public [28].

Today, the new generation of dental technicians in Taiwan are trained through the formal education channel, and school education has replaced the traditional Master-Apprentice system. Many participants in this study showed similar characteristics, such as graduation from junior college, younger age, lower seniority, and scarcity of employers, possibly because the participants were mostly graduates of formal dental technology training institutions and were attending seminars for continuing education credits. The results of the study also demonstrated that those with a professional degree had a lower intention to leave the profession than those with a high school vocational education (traditional Master-Apprentice system). Some traditional apprentice dental technicians were trained on-the-job for university degrees in non-dental fields (that is, oral hygiene), in order to qualify for the national examination. This might explain why university degrees did not have statistically significant influence on the analysis of the intention to leave the profession.

There were 2094 dental technicians and 1068 laboratories in Taiwan at the time of this study [10]. These numbers indicated that in addition to those employed by dental care institutions, many dental laboratories were solely run by the responsible person without any employees. In addition, the participants' reluctance to let their employers discover that they had assisted in inviting their colleagues to participate in this study might also be a reason for the small number of employers in this study sample. In the early days of Taiwan's dental technology industry, it was mostly family-run, Master-Apprentice system inheritance, and workshop-style. In the face of the reduction of patient visits and medical insurance costs, dental technicians adopted the business model of smaller profits and quicker turnover and coordinated with the dentists’ schedule, which often led to excessive working hours [29]. In this sample, 81.7% of participants reported a maximum monthly salary of 40,000 NT dollars, indicating that most earned a yearly income lower than the median of all employees and in the third decile of all health professionals in 2019 [30]. Further investigation focusing on the type of payment and its potential effects on dental technicians are encouraged.

In addition, most participants (95.5%) reported excessive weekly working hours, which was higher than most of the Organization for Economic Co-operation and Development countries in 2019 [31], as well as the average weekly working hours of health professionals in Taiwan [32]. Working overtime is an occupational health issue that has recently emerged in East Asian countries and has also attracted serious attention in Taiwan [33–35]. Long working hours have been found to adversely affect many risk factors of overwork-related diseases such as cardiovascular and cerebrovascular diseases [36]. The current study revealed that dental technicians are working overtime, highlighting the need for the development of policies or strategies to improve working conditions and prevent overwork-related diseases [35].

Similar to previous studies [2, 3], we observed that most participants agreed that DDTs increased the time efficiency and competitiveness of the digital workflow. However, fewer (33.8%) participants agreed that DDTs might increase their income, and more participants agreed that DDT tools and materials are expensive (Fig. 1). Although CDTs were reported as the main job duties, they only represented the current job scope. Young dental technicians frequently changed jobs, and in recent years, dental technology institutions and universities have incorporated digital dental technology into their curriculum. The digitalization of dental technology was found to be widespread in Taiwan, with most participants (75.1%) having learned at least one type of DDT; CAD/CAM was the most frequent type. Participants viewed DDT positively and accepted its advantages, which possibly improved their job satisfaction.

Compared to the norms of occupational burnout [23], dental technicians in this study showed higher personal and work burnout, along with lower over-commitment. Furthermore, their job satisfaction was moderate and lower than the high job satisfaction reported in a previous study among UK dental technicians [12]. Among Taiwanese dental technicians, 32.9% and 28.2% reported the intention to leave the organization and profession, respectively. The main predictors of turnover intentions in this study were identified as geographical area of workplace, work burnout, and job satisfaction; these results were consistent with those of previous studies in healthcare [18–22, 37]. The introduction of new machines such as CAD/CAM and oral scanners has led to automated production and shortened the working hours of technicians. In recent years, some dental technology institutions have shown a trend of medium-sized or large-scale development [28]. To the best of our knowledge, many participants in this study came from two large dental laboratories in Taoyuan and Taichung, which might explain the regional differences in turnover intentions presented in this study. Since some dental laboratories have begun to undertake overseas orders in recent years [28], globalization may increase cross-national business orders and change the business strategies of dental laboratories. Further studies related to the business model and organizational commitment are recommended [38, 39].

There are several limitations in this study. First, most participants were young, junior, and employees, and a potential sample bias could have existed. Second, owing to the adopted sampling and surveying method, this study could not calculate the response rate. Third, different collection method (hard-copy vs. online) may lead to different response quality. Fourth, the cross-sectional design could not lead to causal inference; the percentage of job position as employer was small, and it may have led to bias in multivariate analysis. Therefore, further investigations using randomized sampling to obtain more representative samples are warranted.

## Conclusions

The present study revealed that most of the young dental technicians were working overtime with lower salary and had moderate job satisfaction. Several of them reported high burnout and turnover intentions. Although the DDT acceptance appeared to increased job satisfaction, the influence was limited. As Taiwan's dental care becomes increasingly specialized, it is crucial to maintain the concept of "teamwork" and retain good and professional dental technicians. Strategies for improving the work environment and occupational health of dental technicians should thus be the focus of future studies.

## Data Availability

The datasets generated and/or analyzed during the current study are not publicly available because of the regulation of KMUHIRB, but are available from the corresponding author upon reasonable request.

## Appendix

See Tables 6, 7, 8 and 9.

**Table 6:** Pearson correlation matrix

	DDT acceptance	Personal burnout	Work burnout	Over-commitment	Job satisfaction	Intention to leave the profession	Intention to leave the organization	
DDT acceptance	Pearson Correlation (r)	1						
	P							
	n	332						
Personal burnout	Pearson Correlation (r)	− 0.138	1					
	P	**0.012**						
	n	328	337					
Work burnout	Pearson Correlation (r)	− 0.118	0.862	1				
	P	**0.031**	**< 0.0001**					
	n	332	335	339				
Over-commitment	Pearson Correlation (r)	− 0.076	0.254	0.262	1			
	P	0.167	**< 0.0001**	**< 0.0001**				
	n	332	337	339	341			
Job satisfaction	Pearson Correlation (r)	0.198	− 0.595	− 0.638	− 0.001	1		
	P	**< 0.001**	**< 0.0001**	**< 0.0001**	0.985			
	n	329	334	336	338	338		
Intention to leave the profession	Pearson Correlation (r)	− 0.226	0.390	0.431	0.178	− 0.468	1	
	P	**< 0.0001**	**< 0.0001**	**< 0.0001**	**0.001**	**< 0.0001**		
	n	327	332	334	336	333	336	
Intention to leave the organization	Pearson Correlation (r)	− 0.185	0.495	0.531	0.092	− 0.623	0.600	1
	P	**0.001**	**< 0.0001**	**< 0.0001**	0.092	**< 0.0001**	**< 0.0001**	
	n	328	333	335	337	334	334	337

Numbers in bold indicate statistically significant differences

**Table 7:** Comparison of job satisfaction according to dental technicians’ demographics and working characteristics

Variables	n	Mean ± SD	Test statistic	*p*	Post-hoc comparison
*Sex*					
Male	211	26.05 ± 7.45	− 0.125	0.901	
Female	125	26.16 ± 7.98			
*Age group (years)*					
20–29	232	26.00 ± 7.86	0.049	0.953	
30–39	79	26.28 ± 6.51			
≥ 40	26	26.27 ± 8.88			
*Highest educational level*					
Senior/vocational high school or lower	21	25.00 ± 7.67	0.678	0.508	
Junior college	198	25.79 ± 7.62			
College or higher	117	26.65 ± 7.61			
*Job position*					
Employer	18	31.72 ± 8.33	3.342	**0.001**	
Employee	313	25.64 ± 7.46			
*Working hours (per week)*					
< 40 ^a^	15	32.07 ± 7.21	4.576	**0.004**	a > b, c, d
40–49 ^b^	162	26.49 ± 7.35			
50–59 ^c^	115	24.91 ± 7.24			
≥ 60 ^d^	40	24.88 ± 9.03			
*Salary (per month, NT dollars)*					
< 23,000	15	26.47 ± 7.80	0.982	0.418	
23,001–30,000	100	24.78 ± 6.73			
30,001–40,000	159	26.49 ± 8.03			
40,001–50,000	33	26.21 ± 5.22			
≥ 50,001	27	27.11 ± 9.78			
*Geographical area of workplace*					
Taipei metro area	93	25.15 ± 7.69	1.064	0.374	
Taichung city	97	25.94 ± 7.77			
Kaohsiung city	86	27.17 ± 7.94			
Taoyuan city	32	25.22 ± 6.88			
Other cities	28	27.25 ± 6.63			
*Work experience (years)*					
< 10	296	25.95 ± 7.46	− 0.852	0.395	
≥ 10	42	27.02 ± 8.67			
*Job duties*					
CDTs only	239	25.60 ± 7.16	1.678	0.188	
DDTs only	48	27.25 ± 7.52			
Both	51	27.27 ± 9.47			

Numbers in bold indicate statistically significant differences. ^a^: < 40 hours; ^b^: 40–49 hours; ^c^: 50–59 hours; ^d^: ≥ 60 hours

**Table 8:** Comparison of intention to leave the organization according to dental technicians’ demographics and working characteristics

Variables	Intention to leave the organization (n, %)		χ^2^	*p*
	Disagree	Agree		
*Sex*				
Male	147 (69.7)	64 (30.3)	1.263	0.261
Female	79 (63.7)	45 (36.3)		
*Age group (years)*				
20–29	148 (64.6)	81 (35.4)	2.702	0.259
30–39	59 (74.7)	20 (25.3)		
≥ 40	19 (67.9)	9 (32.1)		
*Highest educational level*				
Senior/vocational high school or lower	13 (61.9)	8 (38.1)	0.570	0.752
Junior college	136 (68.3)	63 (31.7)		
College or higher	75 (76.9)	40 (34.8)		
*Job position*				
Employer	15 (78.9)	4 (21.1)	1.368	0.242
Employee	205 (65.9)	106 (34.1)		
*Working hours (per week)*				
< 40	12 (80.0)	3 (20.0)	1.351	0.717
40–49	106 (65.4)	56 (34.6)		
50–59	76 (66.7)	38 (33.3)		
≥ 60	26 (65.0)	14 (35.0)		
*Salary (per month, NT dollars)*				
< 23,000	12 (80.0)	3 (20.0)	8.252	0.083
23,001–30,000	56 (56.6)	43 (43.4)		
30,001–40,000	113 (71.5)	45 (28.5)		
40,001–50,000	24 (72.7)	9 (27.3)		
≥ 50,001	18 (62.1)	11 (37.9)		
*Geographical area of workplace*				
Taipei Metro Area	64 (68.8)	29 (31.2)	10.358	**0.035**
Taichung city	67 (71.3)	27 (28.7)		
Kaohsiung city	59 (68.6)	27 (31.4)		
Taoyuan city	15 (42.9)	20 (57.1)		
Other cities	19 (70.4)	8 (29.6)		
*Work experience (years)*				
< 10	193 (65.9)	100 (34.1)	1.444	0.230
≥ 10	33 (75.0)	11 (25.0)		
*Job duties*				
CDTs only	159 (66.5)	80 (33.5)	0.153	0.926
DDTs only	34 (69.4)	15 (30.6)		
Both	33 (67.3)	16 (32.7)		

Numbers in bold indicate statistically significant differences

**Table 9:** Comparison of intention to leave the profession according to dental technicians’ demographics and working characteristics

Variables	Intention to leave the profession (n, %)		χ^2^	*p*
	Disagree	Agree		
*Sex*				
Male	154 (73.0)	57 (27.0)	0.309	0.579
Female	87 (70.2)	37 (29.8)		
*Age group (years)*				
20–29	167 (72.6)	63 (27.4)	1.833	0.400
30–39	57 (73.1)	21 (26.9)		
≥ 40	17 (60.7)	11 (39.3)		
*Highest educational level*				
Senior/vocational high school or lower	11 (55.0)	9 (45.0)	9.334	**0.009**
Junior college	155 (77.5)	45 (22.5)		
College or higher	73 (64.0)	41 (36.0)		
*Job position*				
Employer	13 (72.2)	5 (27.8)	0.002	0.962
Employee	223 (71.7)	88 (28.3)		
*Working hours (per week)*				
< 40	11 (73.3)	4 (26.7)	0.141	0.986
40–49	114 (70.4)	48 (29.6)		
50–59	83 (72.2)	32 (27.8)		
≥ 60	27 (71.1)	11 (28.9)		
*Salary (per month, NT dollars)*				
< 23,000	10 (66.7)	5 (33.3)	3.477	0.481
23,001–30,000	69 (69.7)	30 (30.3)		
30,001–40,000	119 (75.3)	39 (24.7)		
40,001–50,000	24 (72.7)	9 (27.3)		
≥ 50,001	16 (59.3)	11 (40.7)		
*Geographical area of workplace*				
Taipei metro area	62 (68.1)	29 (31.9)	18.431	**0.001**
Taichung city	79 (83.2)	16 (16.8)		
Kaohsiung city	63 (73.3)	23 (26.7)		
Taoyuan city	16 (45.7)	19 (54.3)		
Other cities	19 (70.4)	8 (29.6)		
*Work experience (years)*				
< 10	209 (71.6)	83 (28.4)	0.025	0.874
≥ 10	32 (72.7)	12 (27.3)		
*Job duties*				
CDTs only	172 (72.3)	66 (27.7)	1.376	0.502
DDTs only	37 (75.5)	12 (24.5)		
Both	32 (65.3)	17 (34.7)		

Numbers in bold indicate statistically significant differences

## Abbreviations

ANOVA: Analysis of variance
CAD/CAM: Computer-aided design and manufacturing
CDT: Conventional dental technology
CI: Confidence interval
DDT: Digital dental technology
OBI: Occupational burnout inventory
OR: Odds ratio
SD: Standard deviation
